# Imitation, Genetic Lineages, and Time Influenced the Morphological Evolution of the Violin

**DOI:** 10.1371/journal.pone.0109229

**Published:** 2014-10-08

**Authors:** Daniel H. Chitwood

**Affiliations:** Donald Danforth Plant Science Center, St. Louis, Missouri, United States of America; Fred Hutchinson Cancer Research Center, United States of America

## Abstract

Violin design has been in flux since the production of the first instruments in 16^th^ century Italy. Numerous innovations have improved the acoustical properties and playability of violins. Yet, other attributes of the violin affect its performance less, and with fewer constraints, are potentially more sensitive to historical vagaries unrelated to quality. Although the coarse shape of violins is integral to their design, details of the body outline can vary without significantly compromising sound quality. What can violin shapes tell us about their makers and history, including the degree that luthiers have influenced each other and the evolution of complex morphologies over time? Here, I provide an analysis of morphological evolution in the violin family, sampling the body shapes of over 9,000 instruments over 400 years of history. Specific shape attributes, which discriminate instruments produced by different luthiers, strongly correlate with historical time. Linear discriminant analysis reveals luthiers who likely copied the outlines of their instruments from others, which historical accounts corroborate. Clustering of averaged violin shapes places luthiers into four major groups, demonstrating a handful of discrete shapes predominate in most instruments. Violin shapes originating from multi-generational luthier families tend to cluster together, and familial origin is a significant explanatory factor of violin shape. Together, the analysis of four centuries of violin shapes demonstrates not only the influence of history and time leading to the modern violin, but widespread imitation and the transmission of design by human relatedness.

## Introduction

**Figure 1:**
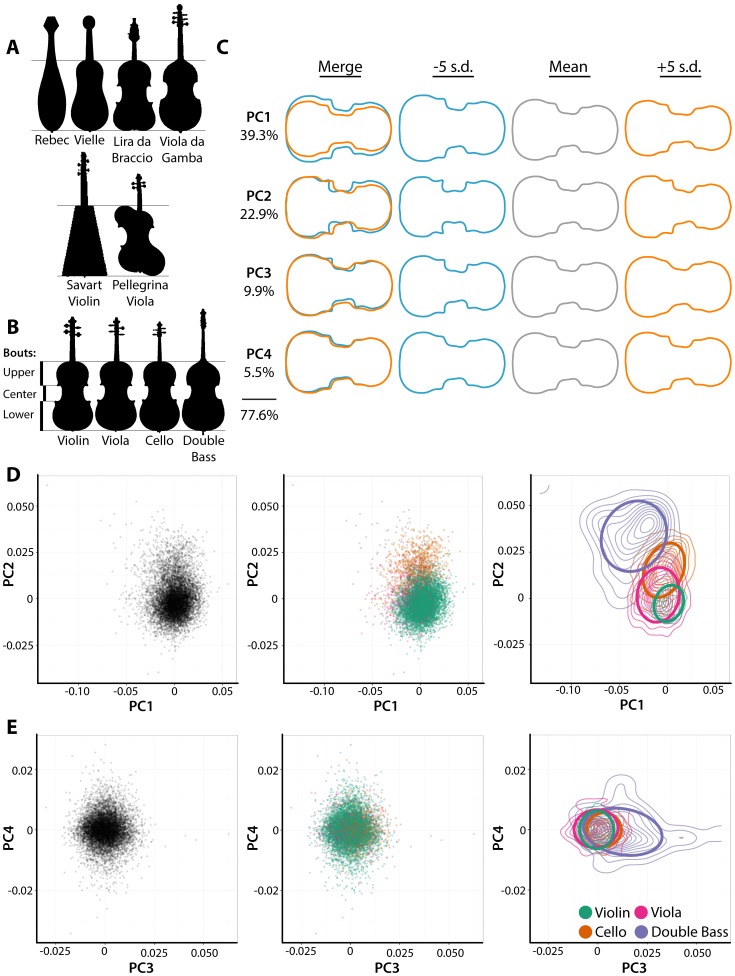
Shape differences between violin family members and close relatives. **A)** Outlines of instruments that likely contributed to violin design (the rebec, vielle, and lira da braccio) and the related viola da gamba family. Outlines of experimental instruments, the Savart violin and Pellegrina viola, are also shown. **B)** Outlines of violin family members. On the left, the upper, center, and lower bout regions are indicated. **C)** Eigenviolins for the violin family morphospace. For Principal Components (PCs) 1–4, violin outlines -5 standard deviations (blue) and +5 standard deviations (orange) along each axis are shown, as well as the mean (gray). Together, the four PCs explain 77.6% of shape variance. Percent variance explained by all PCs and PC values for each instrument can be found in supplemental information. **D–E)** Scatter plots of **D)** PCs 1 and 2 and **E)** PCs 3 and 4. Plots on the left display all instruments and middle plots overlay instrument type by color. Right plots indicate the distribution of each instrument type as a contour plot, upon which is overlayed 95% confidence ellipses. Violins, teal; violas, magenta; cellos, burnt orange; double bases, lavender.

Members of the violin family, their progenitors, relatives, and modern experimental instruments exhibit a remarkable diversity of body shapes (**Fig. 1A–B**) **[1]–[3]**. Some instruments that may have inspired the first violins produced by 16^th^ century Brescian luthiers include the drop-shaped rebec, the box-like vielle (Medieval fiddle), and the lira da braccio, the shape of which resembles modern violins but with a broader base (often heart-shaped) **[4]**. These instruments have distinct timbres and projection compared to the modern violin. Although differences in shape between these instruments are large, they are confounded with a number of other instrument properties, and it is difficult to disentangle the contribution of each attribute to the overall acoustical performance of an instrument.

Indeed, body shape may have little influence over the acoustical properties of modern violins compared to other traits. Although modern violins do vary in the details of their body outlines, shape does not vary as conspicuously as other factors, such as arching patterns, thickness distribution, and wood properties, nor attributes that can be easily changed, such as neck length and angle, bridge design, sound post placement, or even the pairing of bow to instrument **[5]–[9]**. It is remarkable the degree to which the characteristic shape of violins has been neglected (and even purposefully ignored) in modern acoustical research. When first studying plate resonances, Félix Savart went so far as to create a flat, trapezoidal instrument to better focus on Chladni patterns (**Fig. 1A**) **[10]**. Schelleng, in his *The Violin as a Circuit* **[11]**, took a similar view of shape as a hindrance, rather than object, of analysis: “The violin family presents many unsolvable problems; its shape and the peculiarities of its materials were certainly not selected with regard to convenience in analysis.”

In this regard, the body outline of a violin is similar to the shape of *f*-holes. The presence of *f*-holes is highly functional, allowing the breathing of air through the resonant cavity and affecting the normal modes of vibration **[11]**, **[12]**. The details of distinctive *f*-hole shapes, however, that are often used to discriminate the instruments of luthiers from each other, likely provide minor contributions to the differences in projection between instruments. Similarly, the body outline is the context within which the normal modes of a violin are patterned and tonal qualities determined, but the subtle differences in shape from one instrument to another likely account for only small differences in acoustical properties. Like *f*-holes, can body shape be used to distinguish the instruments from different makers? Because the morphological details of body outlines are largely free from functional constraints, what can they tell us about the relationships between luthiers, their influences, and the evolution of complex shapes over time?

Here, the outlines of greater than 9,000 members of the violin family, representing the most prominent luthiers over 400 years of violin making, are morphometrically analyzed. The shapes of violins, violas, cellos, and double basses are first compared. Linear discriminant analysis (LDA) fails to resolve a majority of violas from violins, revealing the compromises that have been made between size, design, and playability to suit the viola's range. Linear discriminants separating violins by luthier are then correlated with time to find specific shape attributes modulated by history. A shape attribute highly correlated with time largely separates early from later luthiers, but also precociously appears in the violins of Antonio Stradivari, preceding his copyists centuries before this element of shape dominated violins of the 20^th^ century. Hierarchical clustering on the averaged outlines of violins produced by prolific luthiers reveals four major clusters of violin shape, one of which acts as an outgroup defining prototypical violins of the Brescian school. Luthiers originating from multi-generational houses tend to cluster together, and family is found to be a significant explanatory factor of violin shape. Together, the outlines of thousands of violins produced over centuries of history demonstrates the gradual evolution of a complex shape subject to historical influences, and the widespread exchange of morphological information through imitation and genetic relationships.

## Results and Discussion

### Differences in shape between instrument types

>9,000 body outlines of violins, violas, cellos, and double basses were obtained from iconography collected from various sources through cozio.com (Tarisio Auctions). Instruments not belonging to the violin family, such as members of the lira da braccio and viola da gamba families, and experimental and oddly shaped instruments, were not included in the analysis (**Fig. 1A**). An Elliptical Fourier Descriptor analysis was used to measure the shape of violin family members (**Figs. 1B, S** **1**) **[13]–[18]** and Principal Component Analysis (PCA) performed to visualize patterns of variance (**Fig. 1C** **; Datasets S1, S2**). The resulting “eigenviolins” describe shape variations among instrument body outlines. Together, the first four principal components (PCs) describe approximately 77.6% of the measured shape variance (**Figs. 1C, S** **2**). PC2, which describes a pattern of shape variance related to the ratios in width of the upper and lower bouts and the proximal-distal placement of the center bout (**Fig. 1C**) separates instrument types by their range (i.e., violins and violas have lower PC2 values and cellos and doubles basses higher PC2 values) (**Fig. 1D**). Double basses, which have viola da gamba-esque tapering shoulders and c-shaped center bouts, show separation from other instruments types by PC1 (describing instrument width) and PC3 (which describes the shallowness of the center bout) (**Fig. 1D–E**).

Schelleng has noted that the problem of scaling instrument size to accommodate different ranges is theoretically possible by maintaining all dimensions and using identical materials **[11]**. Practically, this is impossible. Because the average human dimensions do not change relative to instrument type, simply building a larger violin-to-scale instead of existing viola, cello, and double bass shapes would significantly impact playability, be limited by player stamina, and potentially increase player injury. This is particularly true for the viola, which ideally would be a larger size and played between the legs to accommodate its range, but because of the tradition of playing on the shoulder, is scaled inappropriately, sometimes compromising tone quality **[19]**.

**Figure 2:**
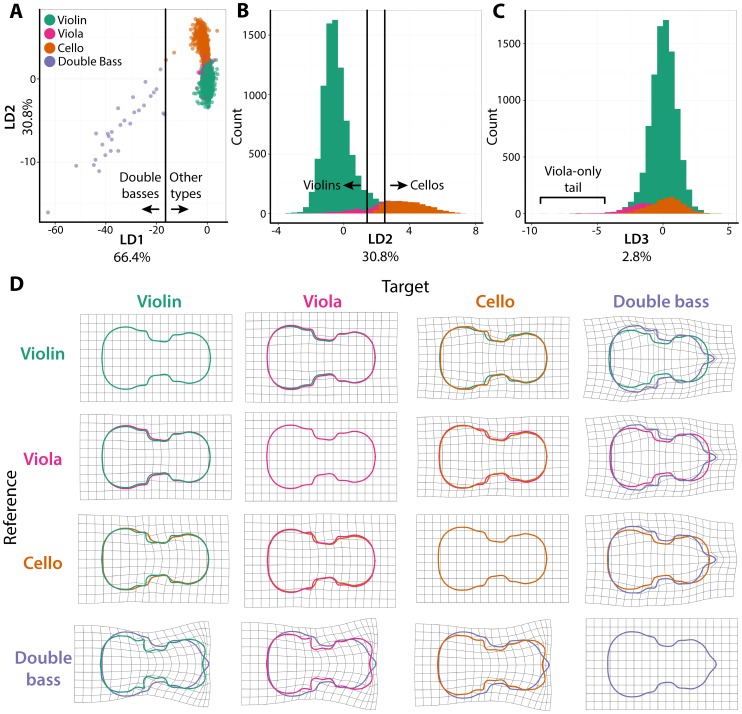
Linear discriminant analysis of violin family members and comparison of outlines using thin plate splines. **A)** Scatterplot of the separation by linear discriminants (LDs) 1 and 2, providing 66.4% and 30.8% of total instrument separation, respectively. Vertical line indicates the LD1 values separating all double basses from other instrument types. **B)** Histogram of LD2 values (30.8% of separation), which largely separate violins and cellos. No cellos have LD1 values less than the LD1 value of the left line, and no violins have LD1 values greater than the LD1 value of the right line. **C)** Histogram of LD3 values (2.8% of separation), which differentiate some violas from violins and cellos. The indicated tail of the viola LD3 distribution does not include violins or cellos. Note: for both panels **B)** and **C)**, double basses are not shown to better focus on violin, viola, and cello distributions. **D)** Pairwise thin plate splines, using grids to show the deformations necessary to transform reference instrument outlines (vertical) into targets (horizontal). Mean outlines of instruments are overlaid and colored to indicate type. Violins, teal; violas, magenta; cellos, burnt orange; double bases, lavender.

To determine the extent that different instrument types are distinguishable from each other, a Linear Discriminant Analysis (LDA), maximizing separation of instrument shapes based on harmonic coefficients, was performed (**Fig. 2A–C** **; Dataset S3**). The first linear discriminant (LD1) explains 66.4% of instrument type separation and mainly differentiates doubles basses, which qualitatively have a distinct and highly variable shape, from other types (**Fig. 2A**). LD2, explaining 30.8% of separation, mainly separates violin shape from that of cellos (**Fig. 2B**) and LD3 explains only 2.8% of separation by type, and is capable of only distinguishing some violas from violins and cellos (**Fig. 2C**). Thin plate splines, which deform a grid so that a reference shape matches a target, can be used to qualitatively analyze the shape characteristics unique to each type (**Fig. 2D**). Violas are wider in the lower bout than violins, whereas in cellos the center bout is displaced more distally and the upper bout narrowed. The shape of double basses is immensely different from other types, with a much wider lower bout, tapered shoulders, and a distally displaced center bout.

To formally determine the degree to which instruments are separable from each other based on body shape, linear discriminants were used to predict instrument class (**Table 1**). Allocation of instrument types reveals that most violins, cellos, and double basses are distinguishable from each other. However, only a fraction of violas (27.8%) are correctly predicted as such, and a majority (62.7%) is wrongly predicted to be violins. The data corroborates common knowledge that, although on average larger than violins, violas are often nearly identical in their shape to them. Nonetheless, a small subset of violas exhibit distinguishable shapes (**Fig. 2C** **;** **Table 1**). Historically, the viola shape and size is non-standardized, and a variety of new shapes, to accommodate playability and reduce injury in players with instruments that are too large (e.g., see the Pellegrina viola, **Fig. 1A**), are currently being designed **[19]**.

**Table 1:** Reassignment of instrument types.

			Reassignment			
Instrument	*n*	Percent correct	Violin	Viola	Cello	Double Bass
Violin	7614	98.4	7493	64	57	0
Viola	601	27.8	377	167	56	1
Cello	1098	79.4	184	42	872	0
Double Bass	31	90.3	0	0	3	28

Reassigned instrument types based on linear discriminants.

### The evolution of violin shape over time

The 16^th^ century was an innovative time in the evolution of Western string instrument shape. As previously mentioned, the violin family likely arose from luthiers in Brescia, using elements from popular string instruments of the 1500s (including the rebec, vielle, and the lira da braccio) (**Fig. 1A**) **[1]–[4]**. Although violin design is always improving and changing, the overall features, and especially the body shape we associate with violins today, arose as early as the mid-16^th^ century. Has the shape of violins remained stagnant since this time, or like other complex morphological phenomena, has it been evolving over the course of four centuries?

To answer this question, I consider only violin outlines for the remainder of this study, which with >7,000 samples, dominates the dataset relative to other instrument types. This dataset is derived from the iconography of auction houses, and so therefore encompasses the most highly desirable violins, but also those of historical importance. An advantage of studying instrument outlines is the increased sampling it allows. For example, works studying the material, physical, or psycho-acoustical properties of Cermonese instruments are often limited to a few or handful of instruments because of the preciousness of the material **[20]–[26]**. Shape, as derived from photos in this work, presents no such sampling limitations.

**Figure 3:**
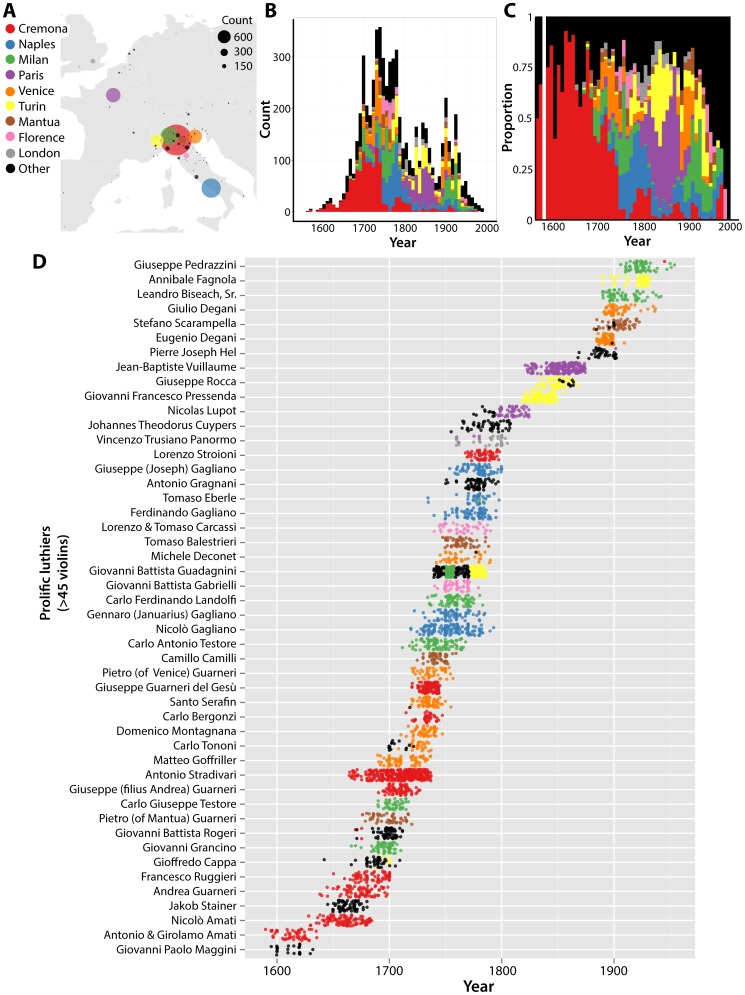
The historical and geographic context of luthiers and their violins. **A)** Geography of violin production in Europe. Overlaid on a map, circle color and location indicates cities of production and size is proportional to the violin output represented in this dataset. **B)** Stacked histrogram of violin production by year. Colors indicate city of manufacture. **C)** Same data as in **B)**, but scaled to show the proportional output of each city by year. **D)** Output of prolific luthiers (with>45 violins in the dataset) over historical time. Points correspond to violins and the year of production, colored by the city of production. Luthiers are organized temporally, by the mean year of their violins represented in the dataset. Cremona, red; Naples, blue; Milan, green; Paris, purple; Venice, orange; Turin, yellow; Mantua, brown; Florence, pink; London, grey; other cities, black.

It is helpful to understand the structure of the dataset with respect to luthier, date of manufacture, and locale, which generally follows the history of violin making in Europe (**Fig. 3**) **[27]–[29]**. Cermonese instruments dominate the dataset, but only until around 1750, after which other Italian schools of violin making rise, including Milan, Naples, Venice, and Turin, as well as those outside Italy, such as Paris and London (**Fig. 3A–C**). Instruments from these cities are often associated with distinct periods of history. Some of this structure is due to the fact that only a handful of luthiers often contribute to a city's output (**Fig. 3D**). MANOVA modeling was used to determine the significance of luthier, year, and city covariates in explaining harmonics coefficients of violin outlines. The final model included luthier and year as significant explanatory variables (**Table 2** **; Dataset S4**). Country was not significant, but only if luthier is the first factor, reflecting the unbalanced design and dependence of factors (i.e., luthiers mostly come from a single city and each city is composed of a small number of luthiers). For these reasons, I chose to ignore city in this particular model and focus on luthiers and time.

**Table 2:** MANOVA results for violin outlines by luthier and year.

Factor	df	Pillai	approx F	num df	den df	p value
Luthier	378	8.0565	1.541	33264	508640	<2.2×10^−16^
Year	1	0.0327	2.185	88	5693	1.57×10^−9^

Results from a MANOVA model fitted for harmonic coefficients as a function of luthier and year effects. Only significant terms were included in the final model.

**Figure 4:**
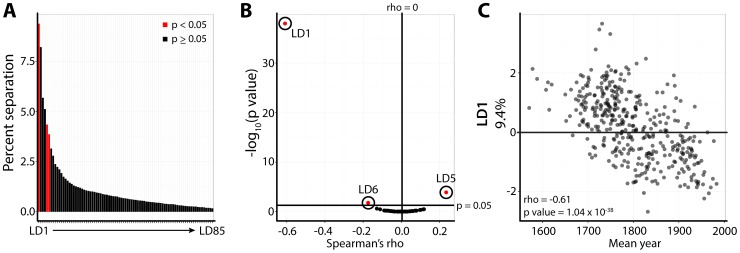
Linear discriminants of luthier correlated with time. **A)** Percent separation contributed by linear discriminants (LDs) 1–85, separating violin outlines by luthier. Averaged LDs by luthier were correlated with the mean year of manufacture for luthiers. Those LDs significantly correlated with time are indicated in red. **B)** Scatterplot of Spearman's rho (x-axis) and -log_10_ p-values (y-axis) for averaged LD values by luthier correlated with mean year of luthier production. Circles, labels, and red indicate significant LD correlation with time. Note that LD1 (9.4% separation) is exceptionally correlated with time. **C)** Scatterplot showing correlation of averaged LD1 values for luthiers with average year of luthier violin production. rho and p values and indicated. All p values shown in this figure are multiple test adjusted across LDs to control false discovery rate (FDR) using the Benjamini-Hochberg (BH) method.

Linear Discriminant Analysis (LDA) was used to separate luthiers by the shape attributes that most distinguish them (**Fig. 4A** **; Datasets S5, S6**). A more important question is what factors influence the shapes of violins that most distinguish their makers. The most obvious factor, available in this dataset, is time. Are the shapes that distinguish individual makers under the control of a higher influence, such as their place in history? To answer this question, linear discriminants for the outlines of violins produced by each luthier were averaged and correlated with the averaged age of instruments for each maker (**Fig. 4B** **; Dataset S5**). After multiple test adjustment, only three LDs were significantly correlated with year, and LD1 (explaining 9.4% of separation between >400 luthiers) exhibited exceptional correlation with time (rho = −0.61, p = 1.04×10^−38^) (**Fig. 4C**).

**Figure 5:**
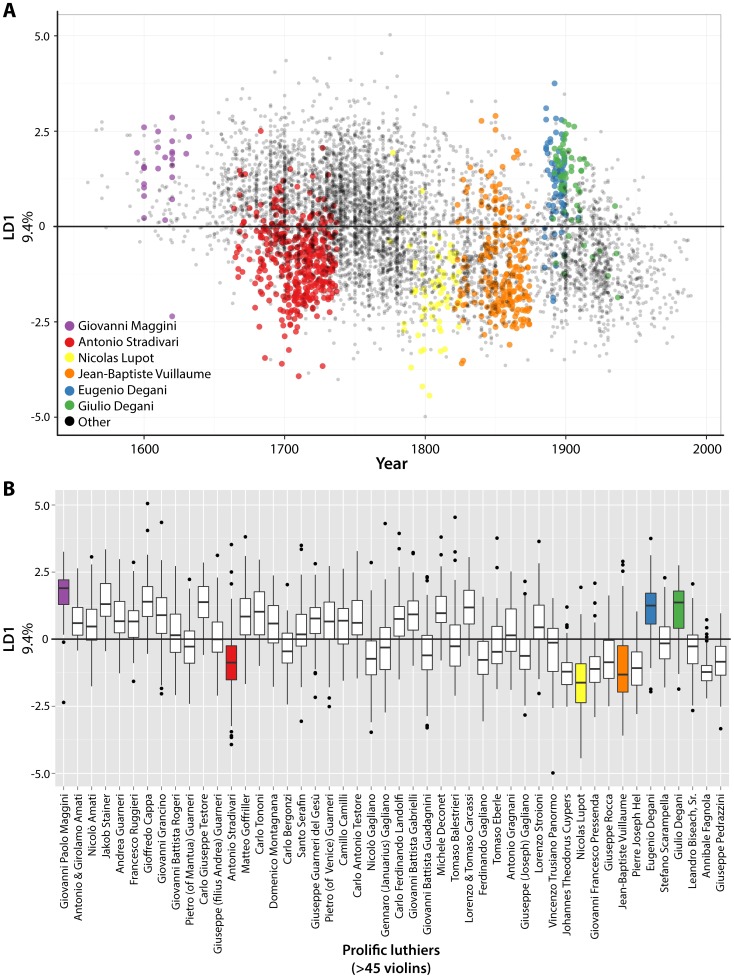
The contributions of luthiers to the correlation of violin shape attributes with time. **A)** Scatterplot showing individual violins with LD1 values (9.4%) plotted against year. Colors indicate violins produced by select luthiers. **B)** Similar to **A)**, showing boxplots of LD1 values of violins produced by prolithic luthiers. Luthiers are arranged temporally along the x-axis by the average year of the violins they produced. See text for details for the relationships of luthiers to each other and known copying of violin design. Purple, Giovanni Maggini; red, Antonio Stradivari; yellow, Nicolas Lupot; orange, Jean-Baptiste Vuillaume; blue, Eugenio Degani; green, Giulio Degani; black, other.

Visualizing LD1 values of individual violins (**Fig. 5A**) and prolific luthiers (defined by>45 violins) (**Fig. 5B**) over time, and comparing with historical accounts of violin making, can offer insights into why this particular shape attribute is temporally modulated. Much of the correlation of LD1 with time seems to be attributable to extreme values before ∼1650 and after ∼1800. Instruments made before 1650 have exceptionally high LD1 values and are almost exclusively derived from Brescian luthiers (e.g., Giovanni Paolo Maggini, **Fig. 5**), representing the first violins. Interestingly, the instruments of Eugenio Degani and his son Giulio Degani at the beginning of the 20^th^ century have anachronistically high LD1 values, perhaps suggesting the Brescian school influenced them. The first luthier to innovate a violin with the opposite extreme of LD1 values was Antonio Stradivari of the Cremonese school. This is noteworthy for two reasons: 1) low LD1 values uniquely define A. Stradivari from his contemporaries, which is important for the identification of violins from this period because of their desirability, and 2) the documented influence of A. Stradivari on subsequent luthiers provides a hypothesis that the low LD1 values in violins after ∼1800 may arise from imitation.

Two of the most famous luthiers that began the trend of low LD1 values after 1800 are the known Stradivari copyists Nicolas Lupot and Jean-Baptiste Vuillaume of Paris (**Fig. 5**). Hart, in his *The Violin: Its Famous Makers and Their Imitators* **[27]**, not only declares N. Lupot the “French Stradivarius” but says, “Stradivari was his idol, and from the fact already mentioned, that he is very rarely found to have followed any other model than that of Stradivari, he would seem to have been aware of his own peculiar fitness for the great master's design.” The violins of J.B. Vuillaume may even have been more influential than Lupot in disseminating the Stradivari shape attribute around the world. His purposeful imitation of Stradivari was profit-driven **[28]**:

*Of all the great Italian masters of violin-making, Stradivari was always his ideal, and by constant study, and cultivation of his own rare natural powers of observation, he acquired such an intimate knowledge and judgment of Stardivari's work in every detail, that he might almost be said to be better acquainted with the maker's instruments than the master himself. Vuillaume soon found the sale of violins, issued as new works without any semblance of antiquity, an unprofitable undertaking, and, recognizing the growing demand in all parts of the world for instruments resembling the great works of Cremona, he determined to apply his great skill as a workman, and his extraordinary familiarity with Stradivari's models, to the construction of faithful copies of the great maker's works. This was the foundation of his success, for the modern copies found a ready sale, and orders poured in upon Vuillaume from all parts of the world.*

Although LD1 is exceptionally correlated with time, it still represents only a fraction (9.4%) of the total separation of violin shape by luthier (**Fig. 4**). Using linear discriminants to predict luthier is a particularly relevant way to detect imitation while using all available separation (**Table 3** **; Dataset S7**). Of the prolific luthiers, Giovanni Paolo Maggini (the only early Brescian in this group) is one of the most discernable, with 78.6% of his instruments being correctly reassigned to him. Instruments by Eugenio Degani, with the anachronistically high LD1 values (**Fig. 5**) are also relatively distinguishable at 63.5% correctly reallocated violins. Nicolas Lupot, the known copyist previously mentioned, has one of the lowest correct reallocation rates (19.5%). But the lowest reassignment among prolific luthiers is Vincenzo Trusiano Panormo, with only 8.2% correctly reallocated instruments. Hart describes both N. Lupot and V.T. Panormo as the “faithful copyists” of A. Stradivari **[27]**:

**Table 3:** Reassignment of luthier identity.

Luthier	N	Correct	Percent correct	Rank
Cuypers, Johannes Theodorus	62	50	80.6	1
Maggini, Giovanni Paolo	56	44	78.6	2
Camilli, Camillo	68	49	72.1	3
Degani, Eugenio	85	54	63.5	4
Cappa, Gioffredo	81	51	63	5
Stradivari, Antonio	410	254	62	6
Lupot, Nicolas	77	15	19.5	43
Rocca, Giuseppe	90	17	18.9	44
Bisiach, Sr., Leandro	65	11	16.9	45
Tononi, Carlo	55	9	16.4	46
Guarneri, Pietro (of Venice)	53	8	15.1	47
Panormo, Vincenzo Trusiano	73	6	8.2	48

Reassigned violin luthier identity based on linear discriminants. Highest and lowest ranking luthiers correctly assigned are provided. The full table can be found in supplemental information.

*Panormo and Lupot share the palm as the faithful copyists of the great Cremonese master. Neither appears to have attempted to create a model of his own; their sole aim was to imitate to their utmost the various patterns of Stradivarius, Guarnerius, and Amati, but they principally confined themselves to those of Stradivarius.*

An analysis of instrument shape by luthier indicates that specific shape attributes are highly correlated with time (**Figs. 4** **–** **5**). Detailed analysis of the discernibility of shapes from different luthiers and historical accounts suggest widespread copying (**Table 3** **; Dataset S7**), particularly of A. Stradivari, contributing to the temporal structure of shape variance and the evolution of the modern violin’s outline.

### Clusters of highly imitated violin shapes

What effect does copying have on the structure of shape variance in violins? Does shape continuously vary, or did copying lead to only a handful of templates? Qualitative assessments of violin shape suggest variations on a theme, or descent with modification **[30]**, of only a few influential outlines. Additionally, the Brescian and Cremonese schools marked an innovative period in violin shape **[29]**:

*Examine and compare the outlines of some of the principal followers of Amati and Stradivari, such as Andreas Guarnerius, Petrus Guarnerius of Mantua, his nephew of Venice, Joseph Guarnerius filius Andreæ, the Rugeri family, Cappa, Carlo Bergonzi, Lorenzo and Joannes Baptista Guadagnini, and you will find that each one struck out a form differing from that of his neighbour, although they were all indebted to the same source for the foundation of their ideas. In fact, from the pioneer Brescians to the latest of the Cermonese, originality of form was ever one of the prominent merits of the many and various makers.*

**Figure 6:**
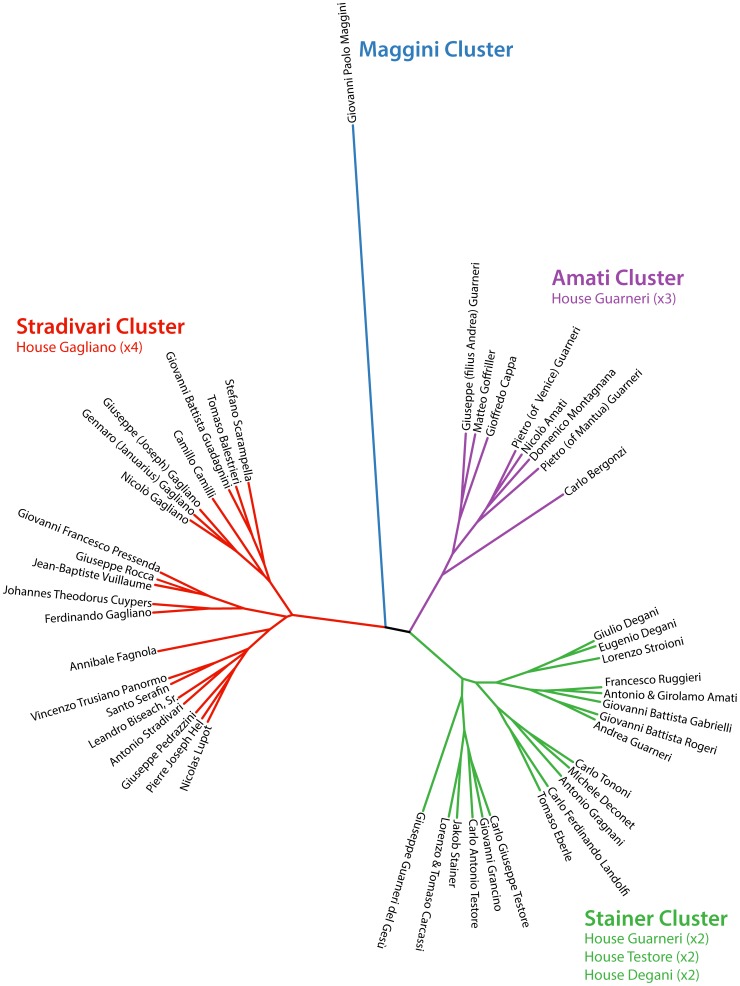
Hierarchical clustering of violin shape. Clustering based on averaged harmonic coefficients by prolific luthier (>45 violins). Four main clusters, named by prominent luthiers they contain, are indicated by color. As discussed in the text, know copyists of Antonio Stradivari cluster with the Stradivari cluster, and often members of the same family cluster together. Members of family houses that cluster together are indicated. Blue, Maggini cluster; red, Stradivari cluster; purple, Amati cluster; green, Stainer cluster.

**Figure 7:**
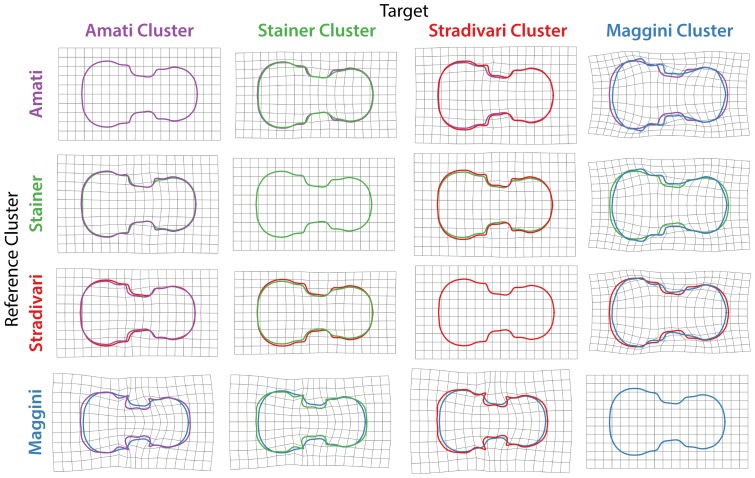
Thin plate splines of major violin clusters. Thin plate splines, deforming grids to transform violins from members of reference clusters (vertical) with those of targets (horizontal), are provided. Averaged violin outlines from prolific luthiers (>45 violins) from each cluster are superimposed and indicated by color. Differences between reference and target outlines have been amplified by a factor of four to better visualize subtle details. Blue, Maggini cluster; red, Stradivari cluster; purple, Amati cluster; green, Stainer cluster.

A more quantitative method to visualize the relatedness of violin shapes is hierarchical clustering. Cluster analysis of averaged harmonic coefficients of violins from prolific luthiers (>45 violins) reveals four major shape groups (**Fig. 6**). These groups have been named “Maggini,” “Amati,” “Stainer,” and “Stradivari” based on their most famous members. The Maggini cluster is particularly interesting. Giovanni Paolo Maggini is the earliest member of the prolific luthiers group and represents the Brescian school. His violins are distantly related to the shape of others, suggesting that the violins of early luthiers had novel attributes that were subsequently lost in modern instruments. Thin-plate splines reveal that Maggini violins have many cello and double bass-like qualities relative to other clusters, with a broad lower bout and shallow center bouts that are distally displaced (**Fig. 7**). Within the Stradivari cluster, the copyists previously mentioned (Nicolas Lupot, Jean-Baptiste Vuillaume, and Vincenzo Trusiano Panormo) can all be found (**Fig. 6**). Stradivari cluster violins are defined by a wide lower bout (**Fig. 7**). Importantly, the existence of the Amati and Stainer clusters suggests other potential copying groups (beyond A. Stradivari) in which luthiers imitate each other (**Fig. 6**). The Amati cluster includes A. Stradivari's student Carlo Bergonzi, who is said to have struck out his own violin outline **[29]**: “Even Carlo Bergonzi was not content with the unsurpassable designs of Stradivari, and seems to have taken the earliest opportunity of assessing his freedom.” Stainer cluster violins are defined by a less defined distal edge of the center bout (**Fig. 7**). Together, there are >4,500 violins in the dataset attributable to prolific luthiers. That such a large number of violins from prominent luthiers cluster in only four groups suggests that violin shape space is not so much continuous as based on variations upon a limited number of copied instrument archetypes.

### Transmission of design by human relatedness

Intriguingly, luthiers that are genetically related often cluster together (**Fig. 6**). For example, four members of House Gagliano are found in the Stradivari cluster, three of which cluster more closely than with any other luthier. Similarly, Eugenio Degani and his son Giulio, uniquely defined by high LD1 values (**Fig. 5**), cluster together in the Stainer cluster, as do Carlo Giuseppe Testore and his son, Carlo Antonio. Members of House Guarneri cluster together, but in two groups, one in the Amati cluster and the other in the Stainer cluster. Remarkably, 54.1% of the>7,000 violins in the dataset are made by a luthier with at least one other relative represented. MANOVA modeling of harmonic coefficients on those violins with at least one other represented family member shows that familial identity is a significant explanatory factor for shape (**Table 4**). Time, and the interaction with family, is also significant, suggesting that each House interacts with historical changes in shape differently.

**Table 4:** MANOVA results for violin outlines by familial identity and year.

Factor	df	Pillai	approx F	num df	den df	p value
Family	24	2.64447	4.7506	2112	81024	<2.2×10^−16^
Year	1	0.09468	3.985	88	3353	<2.2×10^−16^
Family:Year	24	0.92761	1.5424	2112	81024	<2.2×10^−16^

Results from a MANOVA model fitted for harmonic coefficients as a function of family, year, and family:year interaction effects. Only significant terms were included in the final model.

## Conclusions

The violin shape, in its various incarnations of instrument type, has been modified for functional reasons. A viola, cello, or double bass is not merely a scaled-up version of a violin **[11]**, but has been adjusted to accommodate the support of a large instrument over the shoulder (viola), playing between the legs (cello), or the arm span of human beings to simultaneously reach the fingerboard and bow (double bass) (**Figs. 1** **–** **2**). Yet, the vast amount of shape variation within violins is as arbitrary as the violin shape itself. The exact curvatures, the placement and widths of the bouts, the details of the corners, the circumscription of the center bouts into the main body can subtly vary, changing shape, but ultimately not affect the acoustical properties of the instrument. With respect to these types of flourishes, violin shape is modulated by time (**Fig. 4**), in a manner affected by the known imitation of luthiers by one another (**Fig. 5** **;** **Table 3**), resulting in a limited number of archetypal, copied violin shapes (**Figs. 6** **–** **7**).

The parallels between the factors responsible for the changes in violin shape over the centuries and the evolution of complex biological shapes are striking. Violin shape is even influenced by the genetic lineages of its makers (**Fig. 6** **,** **Table 4**). In this sense, shape behaves as a neutral trait. One might easily imagine radically different, but acoustically equivalent, forms of the violin had the whims of the original Brescian luthiers been different. It is not hard to imagine that during long years of apprenticeship within a workshop (which often followed family lines) that peculiarities in the design and shape of instruments, transmitted luthier-to-apprentice, would arise, not unlike genetic drift. The process of creating the outline, whether adhering strictly to a pre-existing mold or pioneering a new shape, is not unlike inheritance and mutation. Despite using molds, Antonio Stradivari nonetheless innovated new shapes, using a method both faithful to previous outlines but with the potential to change **[29]**:

*Hence the question arises, how did he succeed in effecting, year after year, the continual and ever-varying modifications of the curves of the outline? It is evident that he drew a fresh design, and made a new mould for each decided change of form, whether of large or small dimensions; but at the same time we think he probably had some simple plan which, by a slight alteration, permitted him to make use of the old mould while retaining a free hand to alter in a greater or less degree the curves, more especially those of the bouts and corners.*

In another sense, shape can be highly adaptive. Jean-Baptiste Vuillaume purposefully studied and copied the Cremonese masters (especially Antonio Stradivari) to increase the desirability of his instruments and meet consumer demand, as did many others luthiers **[28]**. Recent studies question whether consumers can tell the difference between old Italian violins and other high quality instruments, but that matters little if Cremonese mimics fetch higher prices and sell **[25]**, **[26]**. Certainly an attribute violin consumers would notice is the subtleties of shape, even if not relevant to quality. Perhaps not so surprising for an object crafted by living organisms, themselves subject to natural laws **[30]**, the inheritance of violin morphology was influenced by mimicry, genetic lineages, and evolved over time.

## Materials and Methods

### Image processing

Outlines of violin family members, and their associated data, were obtained from the website cozio.com (Tarisio Auctions). Care was taken to select only those images photographed from a strictly top-down orientation. In ImageJ **[31]**, macros were used to pre-process photos, using built-in “Find Edges”, “Make Binary”, and “Fill Holes” commands. 9,898 pre-processed images can be found in the “Database” section at chitwoodlab.org. The neck of the instrument, button, and other accessories attached to the lower bout were separated from pixels belonging to the body of the instrument by hand before analysis. The resulting violin body was selected and saved as a separate file.

### Elliptical Fourier Descriptor analysis

Binary violin silhouettes were converted to chain code using the program SHAPE **[14]**, **[15]**. The chain code file from SHAPE (.chc file) was then imported into the Momocs package in R **[16]–[18]**, **[32]** using the chc2Coo function and centered with the Coo.center function. Before performing Elliptical Fourier Descriptor (EFD) analysis **[13]**, an estimation of the number of harmonics and smoothening iterations to perform was undertaken. Qualitative analysis of the ability of different numbers of harmonics to recapitulate shape was performed using the hqual function. I was concerned about the ability of the estimated outline to accurately capture the shape of corners of the center bout. The difficulty in capturing this aspect of violin shape was revealed using the hquant function (**Fig. S1A–B**), which plots deviation as a function of points along the violin's outline. I chose to perform the EFD analysis with 22 harmonics and 10 smoothening iterations. This may seemingly be overpowered, as 22 harmonics capture much more than 99% of total power (as determined using the hpow function) (**Fig. S1C**), but outline artifacts were not observed and the corners were adequately recapitulated. EFD analysis was performed using the eFourier function, with which outlines were normalized for rotation, translation, size, and orientation using the first ellipse. Harmonic coefficients from the resulting Coe object were then used for subsequent statistical analyses.

### Statistical analyses and visualization

All statistical analyses were performed in R **[32]**. Principal Component Analysis (PCA) was performed using the Momocs **[18]** pca wrapper function on the resulting Coe object. Eigenviolins along PC axes were visualized using the PC.contrib function. Linear Discriminant Analysis (LDA) on harmonic coefficients was performed using the lda function from the MASS package **[33]**. The predict function (stats package) and table function (base package) were used (dependent on MASS) to reallocate instruments by type or luthiers using the linear discriminants. Hierarchical clustering was performed on harmonic coefficients using the clust wrapper function in Momocs, which uses dist and hclust functions as well as phylo.plot from the ape package **[34]**. A MANOVA was performed on harmonic coefficients using the manova function (stats package). Models were selected by backwards selection, comparing models with and without a term and removing the least significant. Final models were tested with a forward selection process to ensure removed terms were non-significant. Factor order was tested as well, and city was eliminated as a factor because of its dependence with luthier and family. Visualization was performed using the ggplot2 package **[35]**. Thin plate splines were visualized using the meanShapes function to average shapes based on the given factor's levels and passed to the tps.grid function in the Momocs package.

## Supporting Information

Figure S1 **Determining an appropriate harmonic number.** **A)** Deviation, normalized to centroid size (y-axis), of best fit outlines given the number of harmonics (indicated by color) from points sampled along the outline (x-axis). Harmonics double in value from 2 to 64. **B)** Similar to **A)** except analyzing harmonic numbers closer to the value that was eventually chosen (22). Note the four major peaks in deviation relative to other parts of the violin outline, which correspond to the corners of the center bouts. Harmonic number was chosen as a balance between accurately capturing corner shape and outline over-specification. **C)** A graph indicating cumulative harmonic Fourier power (y-axis) for a given number of harmonics (x-axis). Points correspond to medians, with maxima plotted as well. 22 harmonics (the number used for the analysis presented in this paper) captures well over 99% of the harmonic power.
(JPG)

Figure S2 **Percent variance explained by principal components (PCs).** A bar graph showing the percent variance explained by each of 85 PCs for a PCA performed on harmonic coefficients of violin family members. The first four PCs illustrated in **Fig. 1** explain 77.6% of all shape variance, with percent variance explained by subsequent PCs quickly dropping.
(JPG)

Dataset S1 **Percent variance explained by principal components (PCs).** For each of 85 PCs resulting from a PCA performed on harmonic coefficients on violin family members, the PC (“PC”), the percent shape variance it explains (“percent”), and cumulative variance explained (“cumulative”) is given.
(TXT)

Dataset S2 **Principal component values and other associated data for violin family members.** A dataset providing instrument IDs (“id”), luthier (“maker”), the type of instrument (“type”), instrument type in which “small violin” and “small cello” levels have been converted to “violin” and “cello” (“general_type”), the original year information (“year”), a year value representing an average of year spans if present (“year_spans”), a year value in which spanned values have reverted to “NA” (“year_no_spans”), the location of production (“city_state”), the name of the instrument if provided (“name”), and 85 principal component values (labeled “Axis” followed by the appropriate number).
(TXT)

Dataset S3 **Linear discriminant values separating violin family outlines by instrument type.** A dataset providing linear discriminant values based on separation by instrument type, including instrument ID (“id”), instrument type (“type”), and linear discriminant (LD) values 1–3 (labeled “LD” with the appropriate number).
(TXT)

Dataset S4 **Harmonic coefficients used to perform MANOVA on shape by luthier and year.** A dataset providing harmonic coefficient values for violin outlines used for MANOVA, including instrument ID (“id”), instrument type (“type”), the original year information (“year”), a year value representing an average of year spans if present (“year_spans”), a year value in which spanned values have reverted to “NA” (“year_no_spans”), the location of production (“city_state”), luthier (“maker”), the name of the instrument if provided (“name”), and appropriately named harmonic coefficients (“An”, “Bn”, “Cn”, Dn” where n is the harmonic number).
(TXT)

Dataset S5 **Percent separation of linear discriminants of instrument maker and correlation with time.** A dataset providing the percent separation of linear discriminants performed on the harmonic coefficients by luthier and correlation with time, including the linear discriminant (“LD”), the percent separation of the LD (“percent”), the cumulative separation of the LD (“cumulative”), Spearman's rho for the correlation of averaged luthier LD values with year (“rho”), the p value of the correlation (“pvalue”), and the false discovery rate adjusted p value based on the Benjamini-Hochberg method (“bh”).
(TXT)

Dataset S6 **Linear discriminant values based on luthier.** A dataset providing linear discriminant values for separation by luthier, including instrument IDs (“id”), luthier (“maker”), the type of instrument (“type”), the original year information (“year”), a year value representing an average of year spans if present (“year_spans”), a year value in which spanned values have reverted to “NA” (“year_no_spans”), the location of production (“city_state”), the name of the instrument if provided (“name”), and linear discriminant values (“LD” followed by the appropriate number).
(TXT)

Dataset S7 **Luthier reassignment based on linear discriminants.** A table providing the reassignment of luthier identity based on linear discriminant analysis of violin outlines, it includes luthier identity (“maker”), the total number of violins sampled for that luthier (“n”), the number of correctly reassigned violins for the luthier (“correct”), the percent correct reassignment rate (“percent_correct”), and the number of violins reassigned to luthiers represented in the dataset.
(TXT)
