# Supplementary figures and images for: Imitation, Genetic Lineages, and Time Influenced the Morphological Evolution of the Violin
Source: PLoS One. 2014 Oct 8;9(10):e109229. doi: 10.1371/journal.pone.0109229 (PMC4189929; doi:10.1371/journal.pone.0109229)

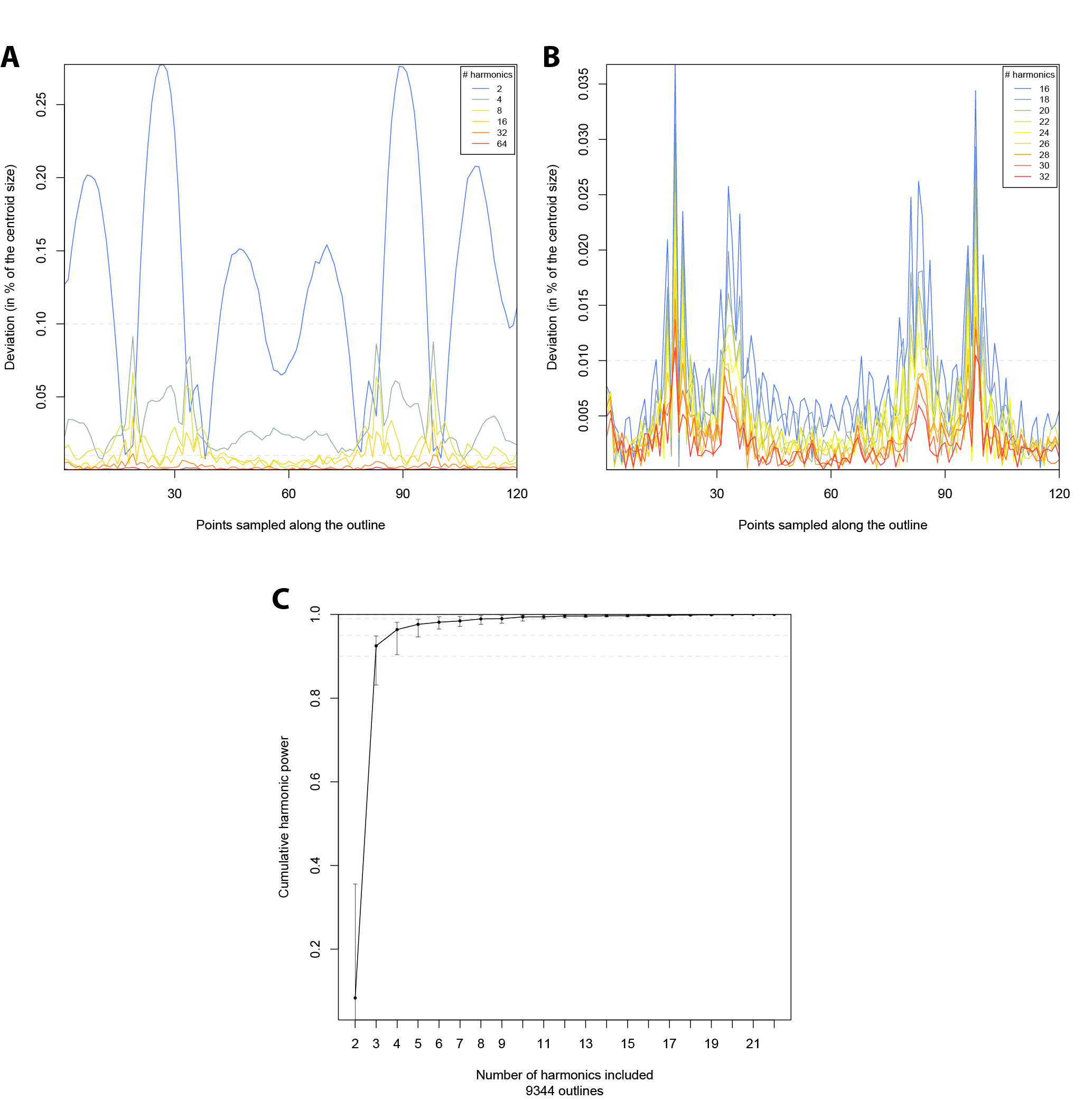

Supplement: Figure S1 — Determining an appropriate harmonic number. A) Deviation, normalized to centroid size (y-axis), of best fit outlines given the number of harmonics (indicated by color) from points sampled along the outline (x-axis). Harmonics double in value from 2 to 64. B) Similar to A) except analyzing harmonic numbers closer to the value that was eventually chosen (22). Note the four major peaks in deviation relative to other parts of the violin outline, which correspond to the corners of the center bouts. Harmonic number was chosen as a balance between accurately capturing corner shape and outline over-specification. C) A graph indicating cumulative harmonic Fourier power (y-axis) for a given number of harmonics (x-axis). Points correspond to medians, with maxima plotted as well. 22 harmonics (the number used for the analysis presented in this paper) captures well over 99% of the harmonic power. (JPG) [file pone.0109229.s001.jpg]

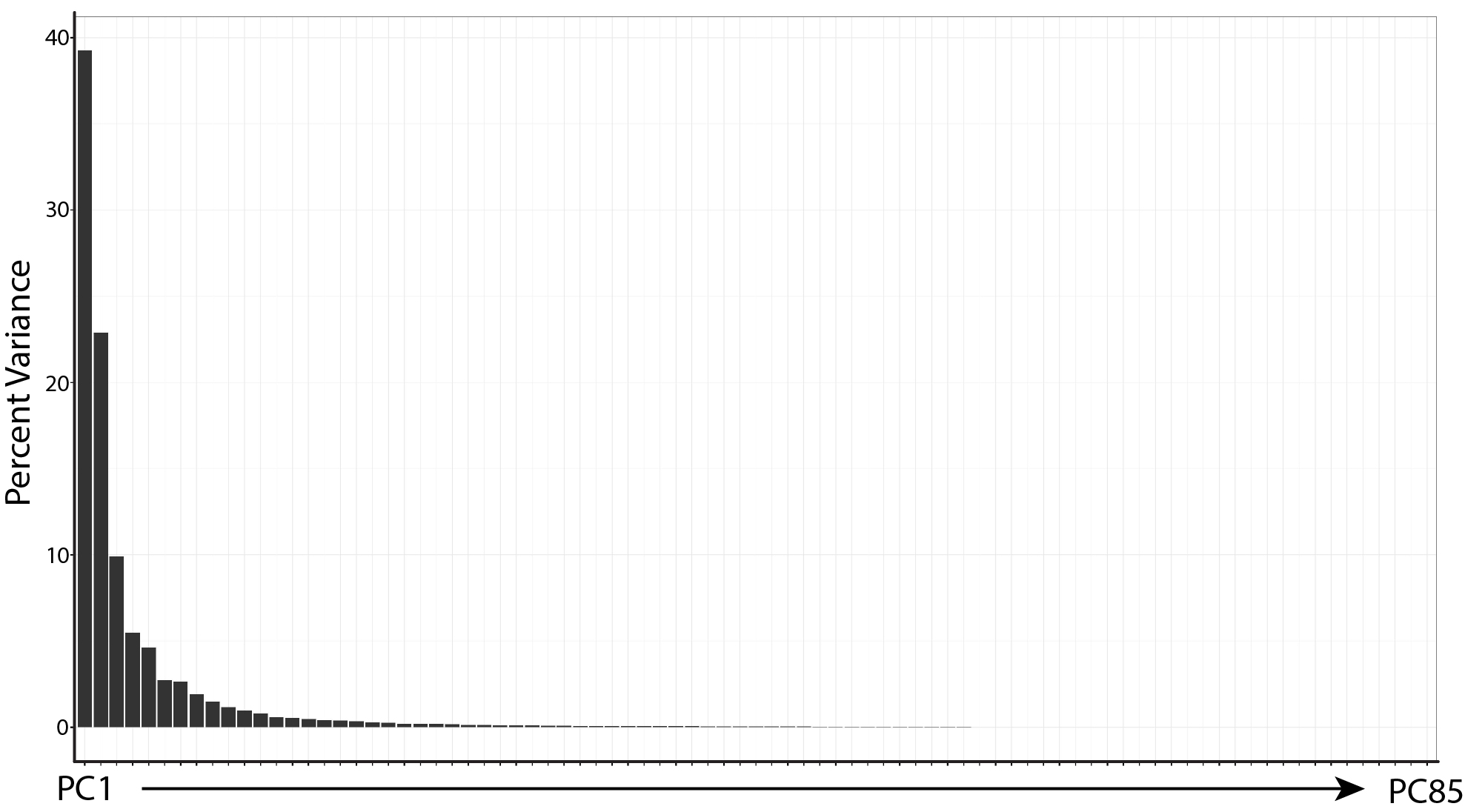

Supplement: Figure S2 — Percent variance explained by principal components (PCs). A bar graph showing the percent variance explained by each of 85 PCs for a PCA performed on harmonic coefficients of violin family members. The first four PCs illustrated in Fig. 1 explain 77.6% of all shape variance, with percent variance explained by subsequent PCs quickly dropping. (JPG) [file pone.0109229.s002.jpg]
